# Genetic analysis of *IRF6*, a gene involved in craniofacial midline formation, in relation to pituitary and facial morphology of patients with idiopathic growth hormone deficiency

**DOI:** 10.1007/s11102-017-0808-8

**Published:** 2017-06-07

**Authors:** Eline Starink, Anita C. S. Hokken-Koelega, Theo J. Visser, Janneke Baan, Robin P. Peeters, Laura C. G. de Graaff

**Affiliations:** 1 Dept. of Internal Medicine, Subdiv. Endocrinology, Erasmus MC, University Medical Center, ‘s Gravendijkwal 230, 3015 CE Rotterdam, The Netherlands; 2 Pediatrics, Subdiv. Endocrinology, Erasmus MC, Rotterdam, The Netherlands; 3 Dutch Growth Research Foundation, Rotterdam, The Netherlands

**Keywords:** Pituitary, Craniofacial, Midline, Genetics, Growth hormone deficiency, IRF6

## Abstract

**Introduction:**

Growth hormone is secreted by the pituitary gland, which forms part of the craniofacial midline. *IRF6* encodes a transcription factor involved in the development of the craniofacial midline and mutations in *IRF6* are known to disturb craniofacial development. Craniofacial and pituitary development are closely related. After whole exome sequencing revealed a new mutation in *IRF6* in a family with Idiopathic Growth Hormone Deficiency (IGHD), we screened the remainder of our IGHD cohort for mutations in this gene and related their genotypes to pituitary and craniofacial morphology.

**Materials and methods:**

We sequenced all coding exons and exon–intron boundaries of *IRF6* in 81 patients with IGHD. We performed a multiplex ligation-dependent probe amplification (MLPA) in order to exclude copy number variations in *IRF6*. We analyzed facial measurements taken from standardized digital pictures of 48 patients.

**Results:**

We found two new variants and eleven polymorphisms. Apart from the new mutation found in the index family (p.Arg233Cys), we found one other new heterozygous missense mutation in *IRF6* (Pro456Ser). p.Arg233Cys was reported as extremely rare in exome databases (1 allele out of 120.852 alleles sequenced), strictly conserved among species and was predicted deleterious by all variant predictor programs. Pro456Ser was predicted to be benign. MLPA did not reveal any exon deletions or duplications in any of the patients.

**Conclusion:**

This is the first report of *IRF6* analysis in an IGHD cohort. We found one new mutation which, based on in silico analysis, could be of functional relevance. However, we did not find any mutations in the other patients. Therefore, we conclude that *IRF6* defects are rare in IGHD patients and further research should focus on new candidate genes.

**Electronic supplementary material:**

The online version of this article (doi:10.1007/s11102-017-0808-8) contains supplementary material, which is available to authorized users.

## Introduction

Growth hormone (GH) is secreted by the pituitary gland, which forms part of the craniofacial midline. Growth Hormone Deficiency (GHD) is the result of insufficient GH secretion, resulting in decreased production of GH-dependent growth factors. The incidence of Idiopathic GH Deficiency (IGHD) is estimated to vary between 1 in 3500 to 1 in 10,000 live births [1–5]. Estimates indicate that between 5 and 30% of idiopathic IGHD case have first-degree relatives with short stature, suggesting a genetic etiology [6].

Although mutations in *GH1* and *GHRHR* can cause IGHD [7–10], the vast majority of patients with IGHD do not carry mutations in these two genes. This suggests that other genes are involved. The identification of these genes is needed to understand the pathogenesis of this complex condition.

Pituitary hormone deficiencies have been associated with defects in the craniofacial midline. Several authors have reported associations between pituitary problems and craniofacial defects [11–17]. Studies investigating hormone deficiencies in larger cohorts of patients with craniofacial clefting show remarkable results. Traggiai et al. studied 19 patients with cleft lip and palate and found GH deficiency in seven of them (37%) [17]. Akin et al. [18] studied 33 patients with median orofacial clefts and found endocrine abnormalities in 22 (70.9%), of which 13 had single and nine multiple hormone deficiencies. Growth hormone deficiency was detected in four of them (12%). There was no relationship between the types of orofacial cleft and endocrine abnormalities. Slavotinek et al. reviewed 31 patients with pituitary duplication. In 19 of them (61%), pituitary duplication was accompanied by cleft palate [19]. Another well-known association between pituitary problems and craniofacial midline defects is called septo-optic dysplasia, defined as the absence of the septum pellucidum combined with hypoplasia of the optic nerve and pituitary dysfunction [20, 21].

Also in patients with Isolated GH Deficiency (IGHD), a broad range of craniofacial midline abnormalities have been described, like hypertelorism, cleft lip and palate and single median maxillary central incisor [22, 23]. The mechanism underlying this association between isolated GH deficiency and craniofacial midline defects is still poorly understood.

Apart from the association between pituitary function and craniofacial features, we also found an association between pituitary morphology and craniofacial features [24].

All these findings are suggestive of an association between craniofacial midline and pituitary development. The mechanism underlying this association has not yet been elucidated.

An important gene involved in craniofacial midline formation is *IRF6* on chromosome 1q32.2. *IRF6* encodes Interferon Regulatory Factor 6, a transcription factor expressed in oral and nasal epithelia. *IRF6* regulates the proliferation and differentiation of epithelial cells during the formation of the craniofacial midline during embryonic development [25]. Mutations in *IRF6* are associated with Van der Woude Syndrome (VWS, OMIM #119300) and Popliteal Pterygium Syndrome (PPS, OMIM #119500). Both syndromes are characterized by a cleft lip and/or cleft palate, which are due to defective craniofacial development. Whereas mutations in *IRF6* cause syndromic orofacial clefting, *IRF6 polymorphisms* are associated with *non-syndromic* cleft lip and/or palate (NSCL/P, OMIM #119530) [26].

Whole exome sequencing (WES) revealed an *IRF6* mutation in one of the families of our IGHD cohort. Since *IRF6* mutations are known to disturb craniofacial development, which is related to pituitary development, we considered *IRF6* a possible candidate gene for IGHD. Furthermore, we hypothesized that variations in *IRF6* might be related to craniofacial morphology. The relation between *IRF6* and pituitary function, pituitary morphology and craniofacial features has not been studied before.

## Materials and methods

### Study subjects

We studied 81 patients with IGHD participating in the Dutch HYPOPIT study, which investigates the genetic causes of idiopathic GH deficiency. These patients had been recruited from the Endocrinology Departments at six university and two non-university hospitals, and had been registered in the Dutch National Registry of Growth Hormone Treatment between 1992 and 2003. IGHD was defined as a peak GH response <6.7 µg/L during Growth Hormone test (mostly arginine tests), or <10 µg/L combined with serum IGF-I < −2 SDS (Standard Deviation Score, according to age- and gender-specific values) and normal serum levels of other pituitary hormones. Exclusion criteria were: GH deficiency of known cause, such as a brain tumor, brain surgery, brain radiation or known syndromes. We obtained approval from the medical ethics committees of all participating hospitals. Informed consent was obtained from all participants and their parents if they were less than 18 years old. 81 IGHD patients who were treated in the participating hospitals and who fulfilled the criteria for IGHD, agreed to participate in the study. All patients received GH treatment. Clinical data of the patients had previously been collected from the Dutch National Registry of Growth Hormone Treatment. *GH1* and *GHRHR* mutations had been ruled out in all patients. MRI reports were available for 67 patients.

### DNA analysis

DNA of 81 patients with IGHD was isolated from whole blood collected in EDTA tubes using standard procedures. We made dilutions of 25 µl/ng of all the collected DNA samples and stored them in a 96-wells plate at −20 °C.

### Whole exome sequencing

DNA samples of the index patient and his first-degree relatives was analyzed by Whole Exome Sequencing: Nimblegen SeqCap EZ Exome v2.0 44 Mb in combination with Illumina Paired-End Library Preparation and 2 × 100 bp Sequencing at 4 Gb per sample. Additional analysis was done with Illumina Human CytoSNP850K SNP arrays.

### *IRF6* Sequencing

After WES revealed the *IRF6* mutation in the index family, we screened the remaining 80 IGHD patients for *IRF6* defects as well. For all patients, we amplified coding exons and exon–intron boundaries by PCR, using primers described by Wang et al. [36]. For exons 3, 4, 8 and 9 we designed new primers using the program Primer3 (reference sequence NM_006147.3). Primers sequences are shown in supplementary Table S1. For PCR, we used the following Qiagen reagents: 10x PCR buffer, 0.2 mM dNTP mixture and 5 units/μl Taq DNA Polymerase. For the primers used to amplify exon 4, we also added 0.5 μl 25 mM MgCl. For primer pairs 1, 2, 5, 6, 7a, 7b, 9a and 9c, we used the standard PCR program. For primer pairs 3, 4, 8 and 9b, we used a Touch Down PCR, lowering the annealing temperature one degree per cycle, during the first ten cycles. PCR reagents and detailed amplification programs are shown in supplementary Tables S2 and S3. After gel electrophoresis, we purified the PCR products using the High Pure PCR product Purification kit (Qiagen) or the Illustra GFX 96 PCR Purification Kit (GE Healthcare) according to the supplied protocols. Sequencing was outsourced to Baseclear Sequencing Services (www.baseclear.com) and carried out using the ABI3730XL sequencer (Life Technologies). The results were analyzed for mutations using Sequencher4.1 (Genecards). For each new variant, the Variant Effect Predictor (www.Ensembl.org) was consulted to predict functional impact. The predictions of this tool are based on the structure and function of the protein and the degree of conservation.

### Multiplex ligation-dependent probe amplification

Copy number analysis was performed using Multiplex Ligation-dependent Probe Amplification (MLPA). We used SALSA MLPA EK1 reagent and P304 *IRF6* probemix according to the protocol of MRC-Holland. We analyzed the results with the program Genemarker (SOFTGENETICS).

### Craniofacial measurements

**Fig. 1:**
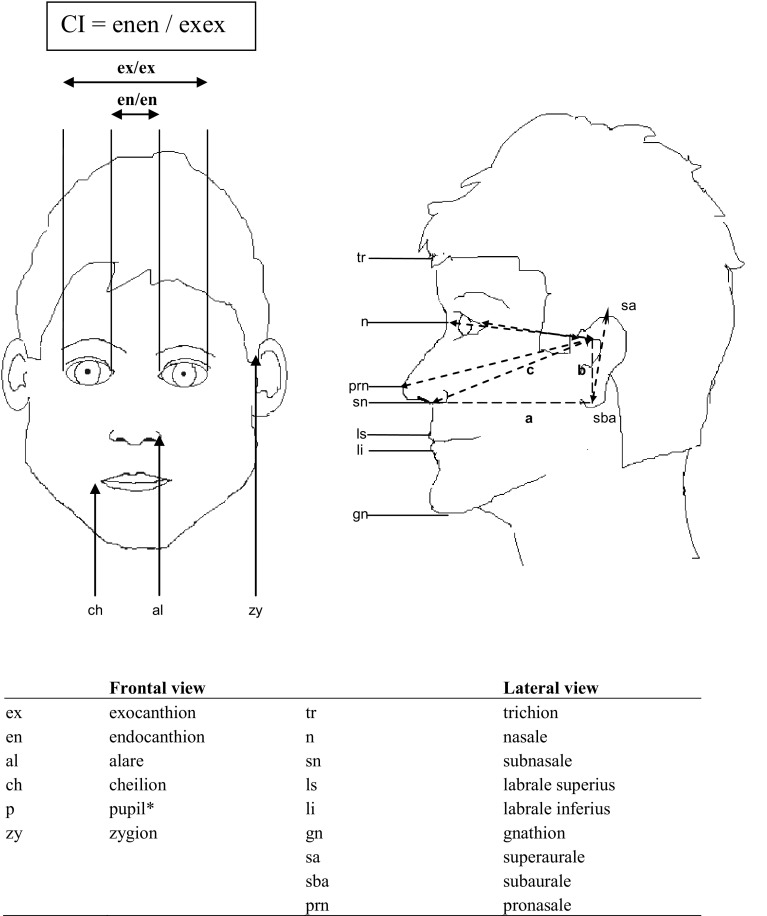
Anthropometric landmarks for frontal facial photographs carried out in our patients

For the current analysis, we measured Canthal Index (CI) from digital photographs taken previously [24]. Frontal and lateral digital photographs were taken under standardized conditions in order to obtain comparable images of reliable quality. Pictures were taken using a Canon^®^ 4.0 Megapixel digital camera. A squared background plate was used to position pictures parallel to the lower extreme of the computer screen, according to the method used by Bishara [27]. Pictures were taken in 48 patients, 4 patients were excluded because of suboptimal quality due to movement artefacts. Adobe Photoshop was used for morphometric analysis. Facial measurements of all pictures were performed by an independent observer (JB). The landmarks we used were those described by Farkas [28] (Fig. 1). Horizontal and vertical lines were drawn and distance between the lines were measured.

### Statistical analysis

We used SPSS 22.0 to analyze craniofacial measurements in relation to growth-related parameters (GH levels during arginine testing, IGF-I SDS and height SDS at start GH). We used one way ANOVA and Chi square analysis to assess differences in clinical parameters according to genotype.

## Results

**Fig. 2:**
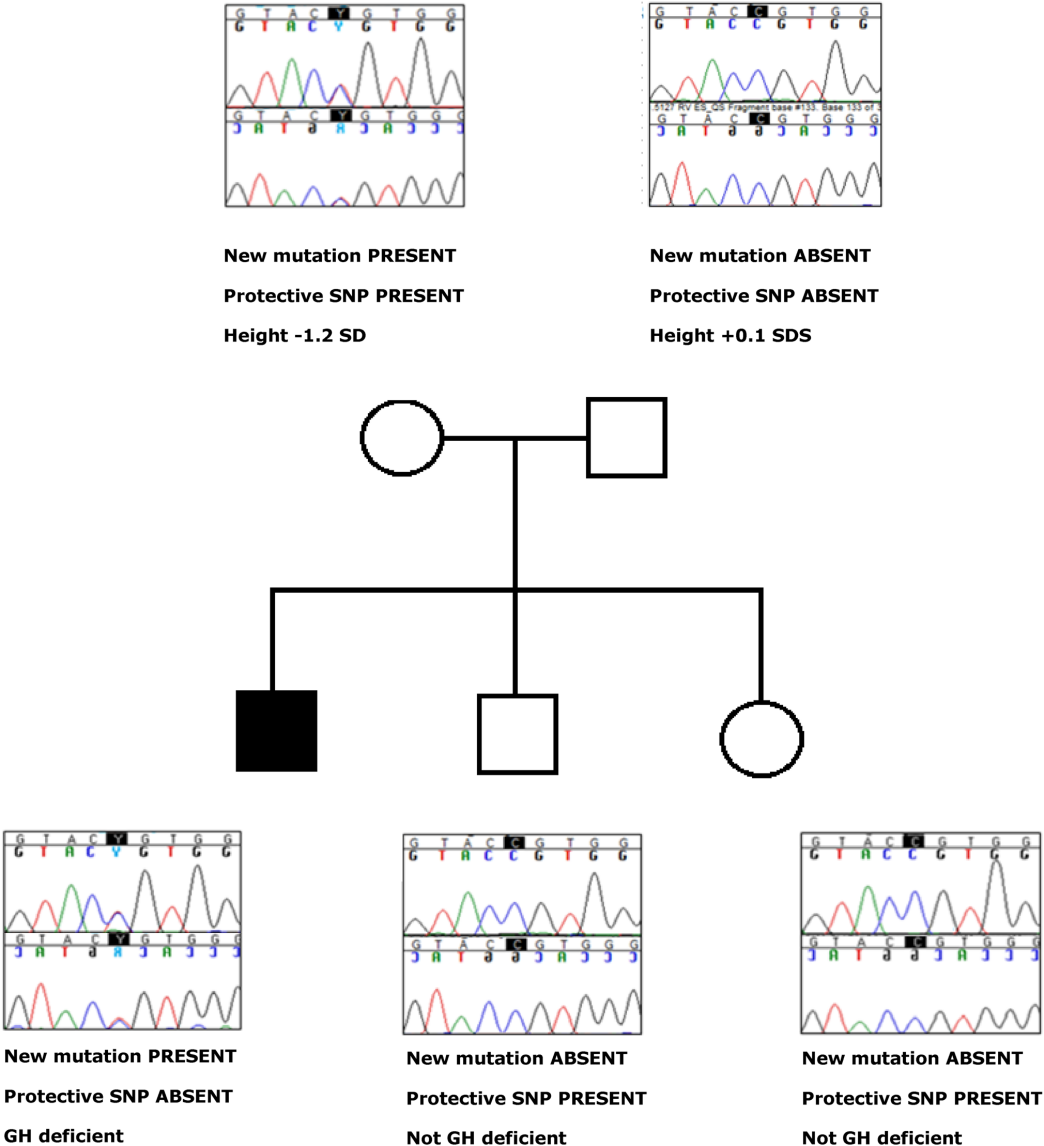
Genotypic and phenotypic data of the family with the new mutation p.Arg233Cys. For each individual, phenotype and presence or absence of the new mutation (p.Arg233Cys) and the protective SNP (p.Val274Ile,rs2235371) is shown

The *IRF6* mutation found in the index family is a heterozygous missense mutation in exon 7, encoding part of the protein binding domain of *IRF6*. The substitution of the C by a T at this position (c.697C>T) results in an amino acid change of Arginine to Cysteine (p.Arg233Cys). The mutation was found in a GH deficient boy with non-consanguineous parents. His father’s height was 182 cm (0.1SD) and his mother’s height was 160 cm (−1.2 SD). His younger brother and sister had growth retardation as well, but they were not GH deficient. The boy was born after 39 weeks of pregnancy and was small for gestational age (2400 and 47 cm). He suffered neonatal jaundice for which he received phototherapy. His arginine test showed a peak GH of 1.7 µg/L, glucagon test showed a peak GH of 2.5 µg/L and his IGF-I SDS before start of GH was −1.4. He did not have any other pituitary hormone deficiencies. At start of GH treatment his height SDS was −2.4. At that time, his bone age was 2 years delayed. He did not have any midline defect and his pituitary MRI was normal. He responded very well to GH therapy; his height SDS increased with 2 SDS and his IGF-I increased from −1.4 to 0.4 SDS. p.Arg233Cys is reported as extremely rare in exome databases (1 allele out of 120.852 alleles sequenced). Multiple species amino acid sequence alignment showed that arginine at AA position 233 is conserved in many different species (overview of species available upon request). The Variant Effect Predictor showed this mutation is deleterious. After finding p.Arg233Cys in the index case, we sequenced both parents, the brother and the sister and found that the mother also carried the mutation (Fig. 2). The mother’s phenotype was normal. However, apart from the new mutation p.Arg233Cys, the mother carried the minor allele of rs2235371. Rs2235371 is a polymorphism known to protect against orofacial cleft formation. Figure 2 shows the genotypes for the new mutation p.Arg233Cys and the protective SNP rs2235371, as well as phenotypic data for the index patient and his first-degree relatives. As seen in this figure, the only person with IGHD is the only person with the new mutation p.Arg233Cys *without* the protective SNP rs2235371 (see also "Discussion" section).

After finding this new mutation in *IRF6* in the index family, we screened another 80 patients with IGHD and we found one more new mutation and 11 known polymorphisms in *IRF6* (Table 1). MLPA did not show any copy number variations. The genotype distributions of the known *IRF6* SNPs in our cohort were consistent with Hardy–Weinberg equilibrium (data not shown) and not significantly different from those in European control cohorts (www.ensembl.org).

**Table 1:** Known and new variants found in the *IRF6* gene in our IGHD cohort

Exon	SNP ID	Position	Impact	Base change	AA change
1	rs34743335	5′upstream		c.-313T>A	
	rs12403006	5′UTR		c.-302A>T	
2	rs2235377	Intronic		c.-75-4A>G	
	rs861019	5′UTR		c.-73T>C	
3	No variants found				
4	rs7552506	Intronic		c.175-5C>G	
5	rs2013162	Exonic	Silent	c.459G>T	p.Ser58=
6	No variants found				
7	**New mutation**	**Exonic**	**Missense**	**c.697C>T**	**p.Arg233Cys**
	**rs2235371**	**Exonic**	**Missense**	**c.820G>A**	**p.Val274Ile**
	rs41303263	Exonic	Silent	c.759T>C	p.Tyr158=
	rs2235373			c.1060+37C>T	
8	No variants found				
9	**New mutation**	**Exonic**	**Missense**	**c.1366C>T**	**p.Pro456Ser**
	rs17317411	3′UTR		c.*451A>G	
	rs75012801	3′UTR		c.*479T>G	

Variants printed in bold are discussed in the “Discussion” section

The other new mutation, p.Pro456Ser was found in a boy diagnosed as GH deficient at the age of 9 years and started GH treatment when his height SDS was −3.8. He did not have any other pituitary hormone deficiencies. Exome databases showed that Pro456Ser was also rare, but the mutation is not strictly conserved among species and predicted to be benign (not damaging). His unaffected father also carried this mutation (data not shown).

**Fig. 3:**
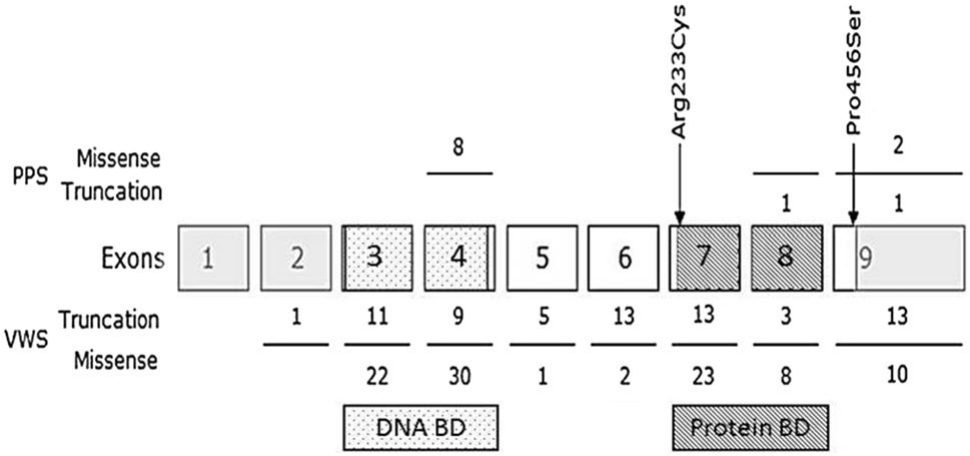
Overview of *IRF6* variants reported in the literature. Numbers above the exons indicate mutations found in patients with PPS, number underneath the exons indicate mutations found in patient with VWS. The *grey boxes* represent non-coding parts of IRF6. Exons 3 and 4 (*dotted*) encode the DNA-binding domain whereas exons 7 and 8 (*striped*) encode the protein-binding domain. The *two arrows* indicate the new mutations found in the current cohort

Figure 3 shows *IRF6* variants previously described in the literature, as well as the new *IRF6* mutations found in the current study. Apart from the two new mutations, we found 11 known polymorphisms. We focused on rs2235371 (V274I), which has been reported to be protective against orofacial cleft formation (see also “Discussion” section). The clinical characteristics of our patient cohort according to rs2235371 genotype are shown in Table 2. Patients carrying the protective minor (A) allele showed a trend towards a less severe phenotype, which was consistent for all clinical parameters measured: they had a higher GH peak during arginine testing, a larger height SDS and a higher IGF-1 SDS at start of GH treatment. Mid-facial hypoplasia, a facial characteristic often seen in patients with severe GHD, was also less often seen in patients with the protective minor allele. None of the patients with the protective allele had orofacial clefts, whereas of the 76 patients without the protective allele, two had orofacial clefts (one female patient had a cleft lip and palate and one male patient had a cleft lip and palate and bifid tongue). Pituitary anomalies on MRI were absent among the patients with the protective allele, whereas they were present in 51% of the patients without the protective allele (0% vs. 51% *p* = 0.017) (Table 3). Although the milder phenotype was clear for all clinical parameters measured, only the difference in MRI results reached statistical significance (*p* = 0.017).

**Table 2:** Clinical data of our patients according to IRF6 rs2235371 genotype

	IRF6 rs2235371			
	G/G	G/A	Total	*p*
Sex				
F/M	24/52	1/4	81	0.63
Height at start GH				
Mean ± SD	−3.2 ± 0.8	−2.9 ± 0.7	81	0.45
Range	(−5.8 to −0.7)	(−3.7 to −2.1)		
Arginin test peak GH				
Mean ± SD	9.5 ± 6.3	11.5 ± 2.7	65	0.53
Range	(0–24)	(8.8–15)		
IGF-I SDS				
Mean ± SD	−3.2 ± 2.4	−2.6 ± 1.5	73	0.55
Range	(−9.0 to 0.9)	(−5.0 to −1.1)		
Birth weight (kg)				
Mean ± SD	3.1 ± 0.6	2.8 ± 0.5	78	0.25
Range	(1.2–4.3)	(2.1–3.2)		
Birth length (cm)				
Mean ± SD	48 ± 3.5	45.5 ± 4.2	60	0.18
Range	(35–52)	(41–50)		
Gestational age at birth (w)				
Mean ± SD	38.9 ± 2.8	38.9 ± 1.3	76	0.94
Range	(32–43)	(37–40)		
Pituitary anomalies on MRI				
**No**	**24 (31%)**	**4 (80%)**	**81**	**0.017**
**Yes**	**39 (51%)**	**0 (0%)**		
**Unknown**	**13 (17%)**	**1 (20%)**		
Midfacial hypoplasia^a^				
Yes	9 (12%)	0 (0%)	81	0.414
No	67 (88%)	5 (100%)		
Midline defect^b^				
Yes	2 (3%)	0 (0%)	81	0.713
No	74 (97%)	5 (100%)		

*GHD* growth hormone deficiency

Significant differences (between people with and without the protective SNP) are shown in bold

^a^Midfacial hypoplasia reported by treating physician, before start of GH treatment

^b^One female patient had a cleft lip and palate, one male patient had a cleft lip and palate and a bifid tongue

**Table 3:** Pituitary morphology in our patients, according to *IRF6* SNP rs2235371 genotype

	rs2235371 genotype	
	G/G	G/A
Normal pituitary	24 (31%)^a^	4 (100%)^a^
MRI	22	4
CT^b^	2	0
Abnormal pituitary (MRI)	39 (51%)^a^	0 (0%)^a^
Classic triad	2	0
Other pituitary anomalies on MRI^c^	2	0
Aplastic AP	1	0
(Partial) empty sella	2	0
EPP, invisible AP	1	0
EPP, AP not described	1	0
EPP, normal AP	8	0
EPP, small AP	8	0
AP small, PP small	6	0
AP small, PP invisible	3	0
AP small, PP and stalk normal	5	0
No pituitary imaging available	13 (17%)	1 (20%)
Total	76	5

*EPP* ectopic posterior pituitary, *AP* anterior pituitary

^a^G/G versus G/A: *p* = 0.017

^b^MRI not available

^c^One patient had an ‘abnormal form’ of the pituitary (not otherwise specified), the other had a hypodense area within the pituitary

Craniofacial measurements were not related to rs2235371 genotype.

As expected, there were slight differences between males and female craniofacial features. Craniofacial measurements according to sex are shown in Table 4.

**Table 4:** Facial measurements of our patients according to sex #=mean of left and right lateral photographs

	N	Mean	SD	Minimum	Maximum	*p*
Canthal index (enen/exex)						
Male	36	0.38	0.04	.31	.55	0.051
Female	15	0.36	0.02	.32	.39	
Total	51	0.37	0.03	.31	.55	
Eye width (exen/exex #)						
Male	36	0.31	0.02	.28	.37	0.047
Female	15	0.32	0.01	.30	.33	
Total	51	0.31	0.02	.28	.37	
Nose width (alal/exex)						
Male	36	0.42	0.04	.36	.55	0.493
Female	15	0.42	0.03	.35	.46	
Total	51	0.42	0.04	.35	.55	
Mouth width (chch/exex)						
Male	34	0.56	0.07	.39	.73	0.240
Female	12	0.59	0.05	.48	.66	
Total	46	0.57	0.07	.39	.73	
Forehead height (trn/trls #)						
Male	23	0.51	0.05	.41	.59	0.054
Female	7	0.55	0.03	.50	.60	
Total	30	0.52	0.05	.41	.60	
Nose length (nsn/trls #)						
Male	23	0.37	0.05	.31	.48	0.225
Female	7	0.35	0.03	.28	.38	
Total	30	0.37	0.04	.28	.48	
Philtrum length (snls/trls #)						
Male	23	0.11	0.02	.09	.15	0.074
Female	7	0.10	0.02	.08	.12	
Total	30	0.11	0.02	.08	.15	
Ear length (sasba/trls #)						
Male	21	0.48	0.04	.42	.56	0.120
Female	6	0.51	0.02	.48	.53	
Total	27	0.48	0.04	.42	.56	

## Discussion

We studied *IRF6* as a new candidate gene for Idiopathic Growth Hormone Deficiency, based on literature data and on Exome Sequencing results of one family of our IGHD cohort. In 81 IGHD patients, we found two new mutations, of which one has a possible deleterious effect.

*IRF6* is a transcriptional activator, which belongs to a family of nine transcription factors that share a highly conserved DNA binding domain and a less conserved protein-binding domain [29]. Defects in *IRF6* can prevent the expression of a large number of genes directly controlled by *IRF6*, listed by Bottia et al. [30].

The new mutation we found in the index family, p.Arg233Cys, is located in the protein-binding domain of *IRF6*. The Arginine residue at position is conserved in many different species (overview of species available upon request), suggesting functional importance. The introduction of a cysteine residue in the protein could cause formation of a disulfide bond, which is likely to affect protein stability. This supports the prediction of Ensembl’s Variant Effect Predictor that the new mutation p.Arg233Cys is deleterious. Apart from the index case, his mother also carried the p.Arg233Cys variant. Interestingly, the mother had a normal phenotype. However, the mother also carried the protective allele of rs2235371 (V274I), whereas the index case did not. Rs2235371 (V274I) affects a highly conserved Valine in the protein-binding domain of IRF6, replacing it with Isoleucine [31]. Although some authors conclude otherwise [32], the vast majority of studies conclude that rs2235371 has an independent protective effect on orofacial cleft formation [33–37]. The only person in this family with GH deficiency was the boy with the new mutation p.Arg233Cys without the protective SNP rs2235371. Our hypothesis is that rs2235371 might also have a protective effect on the IGHD phenotype in this family. Although the functional impact of p.Arg233Cys remains to be confirmed, its effect might be counteracted by the protective rs2235371 allele. Further research should include functional analyses to characterize the effect of the new mutation, also in combination with the protective SNP.

Defects in the craniofacial midline are associated with deficiencies of pituitary hormones, like GH [11–17]. GH deficiency was found in 37% of patients with cleft lip and palate [17]. Although one might expect to find combined pituitary hormone deficiencies in patients with midline defects, Akin et al. [18] found *isolated* pituitary hormone deficiency in 13 of 33 patients with median orofacial clefts. GH deficiency was detected in four of the patients (12%). The mechanism underlying the association between craniofacial midline defects and GH deficiency has not yet been elucidated.

In our study, we found associations which, prudently, might be interpreted as a protective effect of the rs2235371 minor allele on IGHD phenotype. This is in line with literature findings showing an independent protective effect of rs2235371 on orofacial cleft formation. In our cohort, patients carrying the minor allele for rs2235371 had a slightly less severe phenotype. This was consistent for all clinical parameters measured: they had a larger height SDS at start of GH treatment, higher GH peak during arginine testing, higher IGF-1 SDS at start of GH treatment, no pituitary anomalies on MRI and less mid-facial hypoplasia (a facial characteristic often seen in patients with severe GHD). However, since patient numbers are small, *p* values were not below 0.05 and therefore we cannot draw any firm conclusions from this.

Miller et al. [38] reported that *IRF6* SNP rs2235371 was related to craniofacial measurements. However, we did not find any association between rs2235371 genotypes and craniofacial measurements.

To our knowledge, this is the first study investigating *IRF6*, a gene involved in craniofacial midline formation, in relation to facial and pituitary morphology of patients with idiopathic growth hormone deficiency. Although the mutation p.Arg233Cys might have functional impact and thus be a rare cause of IGHD, the majority of patients in our cohort did not have any defects in *IRF6*. We therefore conclude that *IRF6* defects are rare in IGHD patients and further research should focus on other genes.

## Electronic supplementary material

Below is the link to the electronic supplementary material.

Supplementary material 1 (DOCX 29 KB)

## Abbreviations

AP: Anterior pituitary
CLP: Cleft lip and palate
CP: Cleft palate alone
EPP: Ectopic posterior pituitary
GH: Growth hormone
GHD: Growth hormone deficiency
HAP: Hypoplastic anterior pituitary
HSDS: Height SDS
IGF-I: Insulin-like growth factor-I
IGHD: Idiopathic GHD
IRF6: Interferon regulatory factor 6
MLPA: Multiplex ligation-dependent probe amplification
NSCL/P: Non-syndromic cleft lip and/or palate
PP: Posterior pituitary
PPS: Popliteal pterygium syndrome
SDS: Standard deviation score
SNP: Single nucleotide polymorphism
VWS: Van der Woude syndrome
