# Supplementary material for: Genetic analysis of IRF6, a gene involved in craniofacial midline formation, in relation to pituitary and facial morphology of patients with idiopathic growth hormone deficiency
Source: Pituitary. 2017 Jun 7;20(5):499–508. doi: 10.1007/s11102-017-0808-8 (PMC5606942; doi:10.1007/s11102-017-0808-8)
Supplement: Supplementary file 1 — Supplementary material 1 (DOCX 29 KB) [file 11102_2017_808_MOESM1_ESM.docx]

**Supplementary data**

Primer sequences and PCR conditions

| Exon | Forward | Reverse |
| --- | --- | --- |
| 1 | 5'- GCT ATC TGG AAA AGG GCG ACA -3' | 5'- CGC GGA GTG TCA TAT TTC TTG G -3' |
| 2 | 5'- AAG ACA ACT AAA GGT GAA TGG GAA T -3' | 5'- CAG CCT TTG TCG CCA GTG TT-3' |
| 3 | **5'-TTT CCC ACC TGG CAC AGC TT -3'** | **5'- TCC AGA AAG GTC TGA TGG TAG AAG AA -3'** |
| 4 | **5'- AAG CAC ATG CTT TGC AGT GG -3'** | 5'- AAG GCT TTC TTG CTT TAT CCA TC -3' |
| 5 | 5'- TTC CCT TGA TTC TCA CTC TTT TT -3' | 5'- CTC CCA CTT GCT AAC AGT CCA G -3' |
| 6 | 5'- TTG CGT TAG TTA TGG GAA TCA C -3' | 5'- AAG TTA GAA AGC AGG ACA GGA AAG -3' |
| 7a | 5'- ATG CTG GTT GAA AGG TGG CT -3' | 5'- TGG GAG CAA CAA GTG ATG GG -3' |
| 7b | 5'- CAG GGC TGC CGA CTC TTC TA -3' | 5'- AGG AAA GCA GGA AGG TGA AAG A -3' |
| 8 | 5'- ATG TGG CTA GTG GCT ATT GTA ATG -3' | **5'- ATG AGG GCT CAA CCC AGA CC -3'** |
| 9a | 5'- CCT CAG GGC CTC TTT GGT CT -3' | 5'- GAA AAG CAA AGT CTG AAG GGT G -3' |
| 9b | **5'- CTT CAA ACC CAG GAG AGC TG -3'** | 5'- CAA TTT CAG GCA CTA CTC CAA TCT -3' |
| 9c | 5'- TGT GAT TCT CCA AAT ATG CCT AG -3' | 5'- GAC TTT GGC ACC CAT TTC TAT T -3' |

**Table S1** *IRF6* primer sequences. Primers shown in bold were designed for this study, the other primers sequences had been designed previously by Wang, X., et al. [36].

| Reagents | µl |
| --- | --- |
| 10x buffer | 2,5 |
| dNTP 2µM | 2,5 |
| Taq polymerase | 0,15 |
| Primer forward 25 mM | 0,5 |
| Primer Reverse 25 mM | 0,5 |
| Milli Q water | 15,85 |
| DNA 25 ng/µL | 3 |

**Table S2** PCR reagents

| Time | Temperature (°C) |
| --- | --- |
| 3 min | 94 |
| 30 sec | 94 |
| 30 sec | 60 |
| 1 min | 72 |
| 4 min | 72 |

25x

**Table S3a** Standard PCR program (used for primer pairs 1, 2, 5, 6, 7a, 7b, 9a and 9c)

| Time  25x  10x, -1°C per cycle | Temperature (°C) |
| --- | --- |
| 3 min | 94 |
| 30 sec | 94 |
| 30 sec | 70 |
| 1 min | 72 |
| 30 sec | 94 |
| 30 sec | 60 |
| 1 min | 72 |
| 4 min | 72 |

**Table S3b** Touchdown PCR program (used for primer pairs 3, 4, 8 and 9b)
